# Anais Brasileiros de Dermatologia: on the eve of its centennial year^⋆^

**DOI:** 10.1016/j.abd.2024.04.001

**Published:** 2024-05-11

**Authors:** Silvio Alencar Marques, Ana Maria Roselino, Hiram Larangeira de Almeida Junior, Luciana Patrícia Fernandes Abbade

**Affiliations:** a Department of Infectology, Dermatology, Imaging Diagnosis and Radiotherapy, Faculty of Medicine, Universidade Estadual Paulista, Botucatu, SP, Brazil; b Department of Clinical Medicine, Division of Dermatology, Faculty of Medicine of Ribeirão Preto, Universidade de São Paulo, Ribeirão Preto, SP, Brazil; c Department of Dermatology, Universidade Federal de Pelotas, Pelotas, RS, Brazil; d Department of Dermatology, Universidade Católica de Pelotas, Pelotas, RS, Brazil

The *Anais Brasileiros de Dermatologia* (ABD), published for the first time in January 1925 as *Annaes Brasileiros de Dermatologia e Syphilographia*, already had as proposal its bimonthly publication, which was not always possible; however, its publication has been occurring uninterruptedly since then.

From 1983 onward, under the direction of Prof. Rubem David Azulay, the ABD definitively consolidated itself as a modern, bimonthly publication with an emphasis on original articles. Since its creation, it has been maintained with the financial support of the Brazilian Society of Dermatology (*Sociedade Brasileira de Dermatologia*, SBD), making use, over time, of the selfless work, in succession, of the Scientific Editors (Chief Editor and Associate Editors), Reviewers and National and International authors. Although it is not the oldest Dermatology journal in the Americas or even in South America, it is certainly one of the most recognized, due to its practically constant advancement, consolidated by its indexing in LILACS since 1981, SciELO since 2003 and in PubMed/Medline since 2009.

The ABD has, philosophically, provided space to infectious and endemic dermatological diseases, either tropical or not, ancient or not, still prevalent, and which affect a significant part of the Brazilian population,^1^, ^2^, ^3^, ^4^ including original subjects and new technologies.^5^, ^6^ It is one of the rare journals with open access and no cost for authors, whether Brazilian or foreign. But there are challenges to be overcome, both in the present and in the future, which must be addressed in the next issues so that they can be discussed and collectively defined, even if controversial. For instance, keeping the journal in print and online? Only the online version? Exclusively in English? In issues or continuous flow? Advance the discussion on the financing of publication fees with shared costs? These are the most pressing questions. And why should they be discussed? It is suggested that readers consult the articles in references 7 and 8, which emphasize the importance of the metrics that evaluate and rank scientific journals and that reinforce the importance of discussing the items suggested above.^7^, ^8^

At present, it is our responsibility as the ABD, SBD, and their Associates to prepare the celebrations of 100 years of publication, which the ABD certainly deserves. And, the certainty that, in addition to the congratulations that the ABD will receive, we also await achievements resulting from discussions on the issues listed above. We invite Associates and the National and International Editorial Boards to share their ideas and suggestions so that the ABD, in its centenary year, is highlighted among contemporary scientific journals in the dermatological universe.

## Financial support

None declared.

## Authors’ contributions

Silvio Alencar Marques: Approval of the final version of the manuscript; drafting and editing of the manuscript.

Ana Maria Roselino: Approval of the final version of the manuscript; drafting and editing of the manuscript.

Hiram Larangeira de Almeida Junior: Approval of the final version of the manuscript; drafting and editing of the manuscript.

Luciana P. Fernandes Abbade: Approval of the final version of the manuscript; drafting and editing of the manuscript.

## Conflicts of interest

None declared.
